# Unmet diagnostic needs in contact oral mucosal allergies

**DOI:** 10.1186/s12948-016-0047-y

**Published:** 2016-09-01

**Authors:** Paola Lucia Minciullo, Giovanni Paolino, Maddalena Vacca, Sebastiano Gangemi, Eustachio Nettis

**Affiliations:** 1 School and Division of Allergy and Clinical Immunology, Department of Clinical and Experimental Medicine, University Hospital “G. Martino”, Messina, Italy; 2 Unit of Dermatology, “Sapienza” University of Rome, Rome, Italy; 3 Section of Allergology and Clinical Immunology, Department of Internal Medicine and Infectious Diseases, University of Bari Medical School, Bari, Italy; 4 Institute of Applied Sciences and Intelligent Systems (ISASI), Messina Unit, Messina, Italy

**Keywords:** Contact oral mucosal allergy, Stomatitis, Cheilitis, Geographic tongue, Oral lichenois lesions, Burning mouth syndrome, Unmet needs, Hypersensitivity reaction, Diagnosis, Patch test

## Abstract

The oral mucosa including the lips is constantly exposed to several noxious stimuli, irritants and allergens. However, oral contact pathologies are not frequently seen because of the relative resistance of the oral mucosa to irritant agents and allergens due to anatomical and physiological factors. The spectrum of signs and symptoms of oral contact allergies (OCA) is broad and a large number of condition can be the clinical expression of OCA such as allergic contact stomatitis, allergic contact cheilitis, geographic tongue, oral lichenoid reactions, burning mouth syndrome. The main etiological factors causing OCA are dental materials, food and oral hygiene products, as they contain flavouring agents and preservatives. The personal medical history of the patient is helpful to perform a diagnosis, as a positive history for recent dental procedures. Sometimes histology is mandatory. When it cannot identify a direct cause of a substance, in both acute and chronic OCA, patch tests can play a pivotal role in the diagnosis. However, patch tests might have several pitfalls. Indeed, the presence of metal ions as haptens and specifically the differences in their concentrations in oral mucosa and in standard preparation for patch testing and in the differences in pH of the medium might result in either false positive/negative reactions or non-specific irritative reactions. Another limitation of patch test results is the difficulty to assess the clinical relevance of haptens contained in dental materials and only the removal of dental materials or the avoidance of other contactant and consequent improvement of the disease may demonstrate the haptens’ responsibility. In conclusion, the wide spectrum of clinical presentations, the broad range of materials and allergens which can cause it, the difficult interpretation of patch-test results, the clinical relevance assessment of haptens found positive at patch test are the main factors that make sometimes difficult the diagnosis and the management of OCA that requires an interdisciplinary approach to the patient.

## Background

The oral mucosa including the lips is constantly exposed to several noxious stimuli, irritants and allergens. However, oral contact pathologies are not frequently seen because of the relative resistance of the oral mucosa to irritant agents and allergens due to anatomical and physiological factors such as the high vascularization that favors absorption and prevents prolonged contact with allergens, the low density of Langerhans cells and T lymphocytes and the dilution of irritants and allergens by saliva that also buffers alkaline compounds [1].

When the reaction caused by the contact of a substance with the oral mucosa is mediated by immunological mechanisms, predominantly Th1 lymphocytes, it can be assimilated to contact dermatitis of allergic physiopathology and should be called allergic contact reaction. If there is no immune mechanism involved, the proper term is nonallergic contact reaction, but terms like irritant/toxic contact reaction could be used to describe the disease [2].

The spectrum of signs and symptoms of oral contact allergies (OCA) is broad. No single pathognomonic or specific clinical picture of OCA exists; the usual elementary lesions comprise: erythema, edema, desquamation, vesicle formation and ulceration, leukoplakia-like lesions, and lichenoid reactions [3].

Clinical signs are frequently less pronounced than subjective symptoms, and patients commonly experience severe functional problems despite only mild mucosal alterations [3].

Patients with no clinically evident lesions may experience burning or paresthesia, whereas other patients may have pain attributable to lichenoid tissue changes or frank oral ulceration [4].

A large number of condition can be the clinical expression of OCA and it is often very difficult or even impossible to distinguish OCA from chronic physical or chemical irritations, irritative contact dermatitis/stomatitis and other types of stomatitis, chronic trauma produced by teeth or fillings in poor condition, irritation caused by the wearing of dentures, parafunctional habits or other types of trauma and signs of disease with oral manifestations [5].

## Clinical entities associated to contact oral mucosal allergies

Different clinical entities may be associated to an OCA. In some of these the allergic origin is established and the relationship with well known allergenic substances has been clearly demonstrated. Other entities recognize a multi factorial origin and a delayed hypersensitivity reaction may be one of the etiological factors involved (Table 1).

**Table 1:** Classification of pathologies of secure or suspected allergic origin

Pathologies of secure allergic origin	Pathologies of suspected allergic origin
Allergic contact stomatitis	Geographic tongue
Allergic contact cheilitis	Oral lichenoid reactions
	Burning mouth syndrome

*Allergic contact stomatitis* is a contact allergic reaction caused by different substances, which cause inflammation of the entire oral mucosa. Lesions are found in the form of erythema, edema, vesicles, bullae, erosions and ulcerations. Oral flavorings, preservatives, and dental materials are common allergens [4, 6]. When the reaction is caused by prosthetic material, we speak of *prosthetic allergic stomatitis* [7]. Allergic contact stomatitis can be associated with cheilitis.

*Allergic contact cheilitis* is a superficial inflammation of the lip that can occur either alone or be associated with stomatitis or perioral eczema. Usually, allergic contact cheilitis is caused by cosmetic and hygiene products. Less frequently, it is caused by dental material contact with musical instruments, topical medicines or food allergens [4, 8].

*Geographic tongue* is a benign, usually asymptomatic disorder involving dorsal surface of the tongue which appears as depapillated areas with leading and folded edges in yellowish or grayish white color and sometimes with unclear borders. The disorder is characterized by exacerbations and remissions with recovering in one area and the appearance in other areas very quickly; thus, it is also called *benign migratory glossitis* [9, 10]. Allergy has been suggested as a major etiologic factor in geographic tongue and nickel sulphate is the most frequent apten found positive at patch test [9, 11].

*Oral lichenoid reactions* (OLRs) are clinical and histological contemporaries of Oral Lichen Planus (OLP) often indistinguishable in manifestations. In contrast to the idiopathic nature of OLP, OLRs are often associated with a known identifiable inciting factor [12]. The presentation of OLR, in the same way as OLP, can be with reticular white patches, papules, plaques, erosions, or ulceration [13]. The etiology of OLRs may represent the oral manifestation of a chronic irritation in some patients or be the clinical result of a delayed hypersensitivity reaction in others. OLRs have been described in response to numerous culprit factors, including antimalarial drugs, oral antidiabetic medication, antihypertensive agents and nonsteroidal antiinflammatory drugs, as well as acrylic resins and metals used in dental practice [5]. Dental amalgam has been the most implicated restorative material in the induction of OLLs, due to the release of mercury [12, 14].

*Burning mouth syndrome* (BMS) is a complex disorder characterized by warm or burning sensation in the oral mucosa without any visible changes or lesions. This condition is probably of multi-factorial origin and can be classified into two forms: primary (essential/idiopathic), the organic causes (local/systemic) cannot be identified, but the peripheral and central neuropathic pathways are involved, and secondary form, determined by local factors, systemic or psychological. A number of triggers, local or systemic, which may be responsible for the sensation of burning mouth have been identified. Local factors include also contact allergens such as dental material and alloys, allergenic foods in hygienic/cosmetic, antiseptics [4, 15–17].

## Etiological factors

**Fig. 1:**
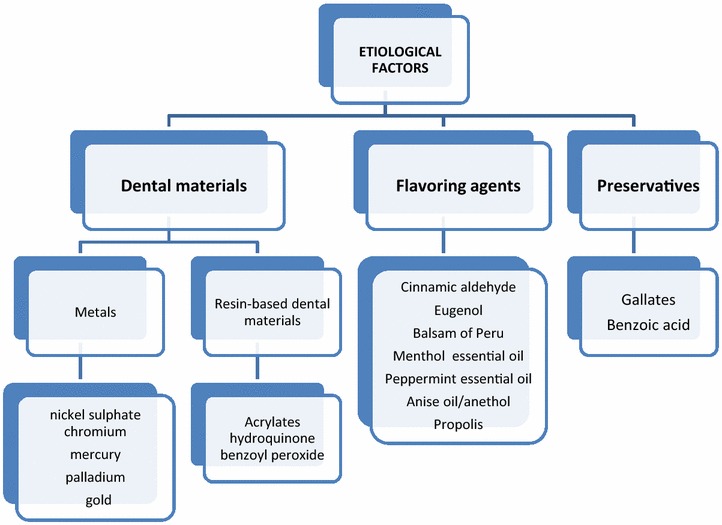
Main etiological factors of OCA

The human oral mucosa is subjected to many pathogens potentially causing a contact allergy. Three types of contact allergy in the oral mucosa can be labelled: dental materials, food and oral hygiene products. The last two factors are involved as they contain flavouring agents and preservatives (Fig. 1).

## Dental materials

Dental products may cause acute, as well sometimes chronic, reactions and problems also to dental personnel because of their occupational exposure. Base-metal dental alloys mainly involved in contact allergy are nickel sulphate, chromium, mercury, palladium and gold. Other materials commonly used in dental restorations including filling, bridges and crowns are (meth)acrylates, composite resins and ethylene amines. Mercury is held in amalgam, a dental alloy used frequently for restoration of teeth for well over 100 years. Mercury can be released as vapour or salt dissolved in saliva during the normal mouth activity like eating or drinking or chewing and the quantity handed out is directly proportional to the one present in the restoration. The others components of amalgam are silver, tin, copper and trace of other metals like zinc. Therefore, the amalgam can locally cause tongue and buccal mucosa lesions like an OLR and the free mercury contained in this type of restoration can cause rise to hypersensitivity reaction [13, 18, 19]. Further, in the work by Raap et al. [20], 28 of the patients have shown allergy reactions to metals used in dentistry. In particular, four patients had positive patch test reactions to mercury. From the study of Dunsche et al. [21] it results that after 20 days of exposition to dental amalgam 96 % of all animals suffered mucosa lesions: 25 % of those had positive patch test to mercury. Also Koch and Bahmer [22] report that 78.9 % patients with OLL were sensitized to mercury.

Nickel is one of the most important metal involved in contact dermatitis and unfortunately its use is very wide in everyday life. Nickel ions, released from nickel-containing alloys used frequently in dentistry, may induce OCA. In a study by Khamaysi et al. [23] in patients who had undergone dental treatment whit oral contact lesions, nickel sulphate was the metal mainly involved with a rate of 13.2 %. On the contrary, in an important meta-analysis that involved thirty studies, it was clear that orthodontic treatment with nickel-containing alloys had no significant effect on nickel hypersensitivity. However, in dentistry it is a base-metal alloy largely used. In the medical literature you also find studies of biocompatibility effects of exposure to base metal dental alloys. One of them employed a three dimensional human derived oral mucosa model to asses this biocompatibility [24]. In another study, it was hypothesized that this kind of human model would provide insights into the mechanism of nickel-induced toxicity. The oral mucosa model treated with nickel–chromium alloy has been compared with one treated with cobalt–chromium alloy and one untreated. The adverse effects increased for the Ni–Cr alloy, proving a Co–Cr enhanced biocompatibility [25]. Lastly, was also studied the release of nickel ions from stainless steel alloys used in dental braces. In this study [26] in 31 nickel-sensitive individuals that were treated with four different stainless steel alloys were searched the amount of nickel ions in saliva and sweet and patch test reactivity for all the alloys. The results showed that small amount of ions were present both in saliva and sweet and none of the nickel-sensitive subjects had positive patch test with the four alloys, indicating that these stainless steel alloys would be safe also in patients with nickel sensitivity. There are also hints in the literature about titanium dental implants. In a study by Flatebo et al. [27], the objective was the histological evaluation of a non-perforated mucosa covered by a maxillary titanium implant with regard to its tissue reaction.

The study included thirteen patients without previous implants. From the histological analysis of the tissues, any sensitivity reaction to titanium implant was proved.

Gold is another metal that can cause a contact hypersensitivity. It is widely used in dentistry as well as in piercing. In dentistry, it is mainly used for the restoration of rear dental arches because in that site the strength is more important than the esthetics. The gold alloys are composed by 80 % of this metal. In several studies it resulted the most common allergen after the nickel [28]. In a study by Vamnes et al. [29], 25 % of patients showed a positive reaction to gold at the patch test and there was a statistically significant correlation between positive tests and presence of dental gold.

Also in Ahlgren’s study [30] there was a statistically significant correlation between positive patch testing to gold in a rate of 30.4 % among the patients involved in the study and the presence of dental gold in a rate of 74.2 % among the previous rate.

In the palladium alloy this metal is present at 75 % and it is known that palladium, in ionic form and at sufficiently high concentrations, has toxic and allergic effects on biological systems.

The allergy to palladium almost always occurs in individuals who are sensitive to nickel [31]. In a recent study by Muris et al. on patients with oral disease, 24.3 % reacted to palladium and 25.2 % to nickel. The patients with palladium sensitization was associated with oral restoration like dental crows and they had lamented OLRs, xerostomia, and metal taste. In conclusion of this study, it was evinced that patients with dental restoration with palladium and oral disease should get themselves checked [32].

Resin-based dental materials are synthetic resins. More precisely, they are self-curing acrylic resins based on polymathic methacrylate.

There may be OCA caused by this kind of dental materials used for fillings or restoration. Tillberg and al. [33], have conducted a study recording time to onset, duration and any reactions after exposition to resin-based dental substances. Of 618 patients observed, 36 were affected by oral lesions, intra and extra-oral, appeared within 24 h after treatment. The patients mainly showed skin problems, oral ulcers and burning mouth. The conclusion of this study was that immediate reactions were more frequently than delayed reactions and they established that such events were not allergic reactions.

Also Kaaber et al. have published 12 cases of allergic reaction to dental resin like burning mouth and stomatitis [34].

It has been studied the potential toxicity of methyl methacrylate in dental use for patients and dental personnel and at least in vitro it is possible to evaluate cells toxicity from this dental material. The observable reactions could be asthmatic symptoms, local neurological symptoms, irritant and local dermatological reactions [35]. As shown by several studies reported in medical literature by authors like Kanerva, contact allergy to (meth) acrylates is most commonly observed in dental personnel. In contrast, this type of contact allergy in patients is less frequent and indeed only case reports can be found in literature [36].

### Flavoring agents

While many studies investigated the role of metals and dental materials in patients with OCA, the involvement of flavoring agents and preservatives were rarely examinated.

Flavoring agents involved in OCA are usually used in food products, skin care products and oral hygiene products as toothpaste and mouthwash. Torgerson et al. found that the most allergenic flavoring was fragrance mix, with a rate of positive reactions of 9.8 %. However, eugenol tested as a single allergen was positive in 0.7 % of cases [4]. Other studies showed eugenol-induced positive reactions in 0, 0.6, and 2 % of patients [37–40]. Balsam of Peru was the second most reactive flavouring agent reported in the study of Torgerson with a rate of 7.2 % [4]. Also cinnamon products can cause oral hypersentivity reactions [41]. However, the real incidence of OCA to cinnamic aldehyde is not known. This oral contact allergy is a rare condition also known as *cinnamon contact stomatitis* [42, 43]. Other flavouring substances, such as menthol or peppermint essential oil, can cause oral contact reactions [44–47].

Cases of stomatitis and cheilitis sometimes combined with loss of taste, have been worldwide associated with exposure to anise oil and/or anethole used as flavoring agents [reviewed in 47].

Propolis also, used as lozenges, solutions or sprays, toothpastes and mouthwashes may cause stomatitis, cheilitis and ulcerations. It can cause also an occupational contact allergy in musicians and people who make stringed musical instruments [48–53].

### Preservatives

The three main gallates are octyl, propyl and dodecyl are responsible of OCA, mainly cheilitis and stomatitis, due to the ingestion of gallates-containig food (such as bakery products) and the use of cosmetics, in particular lipstick [4, 39, 54–56].

Another preservative responsible of oral OCA is benzoic acid, with a rate of positive reactions reported between 3 and 11 % [4, 38, 41, 56].

## Diagnostic tools

### Objective examination

The personal medical history of the patient is helpful to perform a correct diagnosis, as a positive history for recent dental procedures. In this regard, also the specific anatomic region of the oral mucosa can help the clinician in a correct diagnostic orientation. The involvement of the lateral tongue and buccal mucosa are more suggestive for OLRs, rather other diseases; for this reason, the sidedness of the lesions (rather than symmetry) favor the diagnosis of OLRs [57]. Furthermore, rarely, OLRs involves gingivae and palate, being less involved by the dental restorations.

In OCA the main diagnostic patterns are the chronic lichenoid pattern and the erythematous/patch pattern; other rare clinical presentations are urticarial lesions, edematous lesions, ulceration and vesicular lesions [58]. In acute contact mucositis, at first, the area is swelling and vesicular, associated with an itching and burning sensation, while in the later stages the mucosa becomes whitish with the clinical findings reported above. The main causes of acute contact mucositis are gloves, latex, toothpastes, and every possible allergen, that came into contact with the mucosa [13]. In these cases, the diagnosis is often clinical and objective (due to the direct relationship between the allergen and the mucosal reaction), without the necessity to perform a biopsy. Finally, sudden rashes involving both the oral cavities and lips associated with itching and burning, are often suggestive of an allergy to chemicals toothpastes, dental floss and chewing gum, because these products act on a wider anatomical area [57].

### Histology

Histology in the more uncertain lesions is mandatory. In this latter case, the presence of eosinophils together with spongiosis, exocytosis of lymphocytes with occasional neutrophils, thickening of the basement membrane region, keratinocyte apoptosis, plasma cells and peri-vascular infiltrate, allow the diagnosis of OCA, excluding lichen planus and/or other disorders. While, a singular histology is seen only in dermatitis due to cinnamon, where a chronic interface mucositis is mixed with lymphocytes, plasma cells, and histiocytes with a peri-vascular lymphoid infiltrate [59]. However in several cases the pathology is not diriment and the correct diagnosis may be made only with the clinical examination and/or with the use of cutaneous patch tests.

### Patch tests

Up to date there is not a standardized consensus regarding the allergen used for the test, however there are series of allergens (under the European consensus), which include several dental materials, as well as other additional allergens [13]. Usually skin testing is preferable to mucosal testing, due to a higher specificity and sensitivity, as well as to the simplicity of the procedure [60]. Besides, in order to perform a test, the concentration of the allergen should be 5–12 times higher in the oral mucosa, if compared to the skin, resulting in more adverse events [13].

Homstrup et al. [60], trying to avoid the routine use of patch tests for all patients with lichen planus like lesions and threatening unnecessary sensitizations in this class of patients, he listed the required points to perform patch tests in contact oral dermatitis, as follows: (1) OLRs or mucositis resistant to treatments; (2) objective and clinical evident relationship between the mucosal lesions and the suspected allergen; (3) absence of symmetry in the lesions. However patch tests can show false positivity and for this reason they are not reliable in the 100 % of cases [13]. In this regard, a careful clinical examination remains the main diagnostic orientation.

The baselines allergens used during the clinical practice regards in Italy about 28 substances, while targeted testing is designated exclusively by the specialist and thus applied to allergens according to profession (according to the sample of the material brought along). Specifically for dental materials, dermatologist conducts epicutaneous testing for certain substances, in cooperation with the dentist. According to Khamaysi et al. the most common contact allergens in OCA are gold sodium thiosulfate (14 %), nickel sulfate (13.2 %), mercury (9.9 %), palladium–chloride (7.4 %), cobalt–chloride (5 %) and 2-hydroxyethyl methacrylate (5.8 %) [7, 23]. At the same time, most common allergens for specific oral diseases are summarized on Table 2.

**Table 2:** Most common allergens for specific oral diseases

Disease	Allergen
Burning mouth syndrome	Potassium dicyanoaurate
Lichenois reactions	Potassium dicyanoaurate
Cheilitis	Aroma mixtures
Stomatitis	Mercury
Gingivitis	Potassium dicyanoaurate
Orofacial granulomatosis	Nickel sulfatehexahydrate
Perioral dermatitis	Cobalt–chloride
Recurrent stomatitis aphtosa	Vanillin

### Unaccepted methodologies

Finally, still unaccepted methodologies in this area, that have been used without real evidence are oral patch tests. Indeed, their effectiveness in the clinical practice is not yet accepted and there are no objective evidence about this diagnostic tool [37].

## Unmet needs

Nowadays the diagnosis of OCA relies, aside an accurate clinical examination, on patch testing and histology [61]. Even though largely used for contact dermatitis, with regards to contact allergies of the oral mucosa, patch testing might have several pitfalls. Indeed, in OCA, the haptens triggering the activation of T cells are often metal ions constantly eroded from metallic materials present in the oral mucosa. These metal ions, under neutral pH conditions, are normally at low concentrations. An ideal patch test should reproduce the metal erosion that occurs in the metal equipment. However, a standard preparation for patch testing for metals typically contains a metal salt with higher metal ions concentrations compared to the condition of the oral mucosa. Moreover, the metal salt is dissolved into an acidic medium, whereas the oral saliva pH is normally neutral [40]. These latter two conditions, in terms of diagnosis, might result in either false positive/negative reactions or non-specific irritative reactions. To overcome these limitations it has been recently proposed, with encouraging results, on mouse models, to use metal nanoballs for patch testing. These metal nanoballs are indeed conceived to mimic the ions release happening in vivo in patients [62].

Another limitation of patch test results is the difficulty to assess the clinical relevance of positive haptens and only the clearance of a reaction after avoiding a contactant may demonstrate the haptens’ responsibility. However, the number of contactants encountered in daily life, particularly in oral mucosa, and the wide chemical complexity of these contactants makes avoidance challenging. [4, 63].

Histology is another important diagnostic tool that can help diagnosis in the case of unclear clinical presentation of OCA. As for patch testing this latter technique can be further improved. The T cell infiltration present in delayed hypersensitivity reactions can be also observed in other chronic clinical conditions. Therefore, in the case of OCA it would be useful to know whether at the site of the lesion, T cells specific for a given hapten are present. To this aim it would be useful to analyse the TCR repertoire of the infiltrating T cells. In pre-clinical models of metal allergy, this has been done with promising results showing that during chromium-induced allergic contact dermatitis, chromium-specific T cells accumulate at the site of inflammation [64].

A recent work by Di Tola et al. using flow cytometry technique demonstrate that in nickel sensitive patients, after oral exposure to nickel, circulating Th and Cytotoxic T cells are significantly increased [65]. With regards to OCA the analysis of peripheral blood population using flow cytometry would be a useful as complementary tool for diagnosis. This would help to characterize the T cell subtypes involved in the allergic reaction and, thereby, find a more personalized and efficient treatment.

## Conclusions

Although not so frequent, OCA might be observed in the daily practice, causing non-rare diagnostic pitfalls. The spectrum of clinical presentations is very wide and delayed hypersentivity mechanism has been demonstrated in only few entities such as allergic contact stomatitis and cheilitis, whereas in the other diseases as geographic tongue, OLRs and BMS contact allergy is one of the possible triggering factors.

The range of materials which can cause an OCA is very broad. In addition to the dental materials that remain for long time in the oral cavity, numerous are the substances that daily come in contact with the oral mucosa though food and oral hygiene products. Therefore, is very difficult to find the culprit substance. The knowledge of patient’s habits and an accurate clinical examination together with patch testing with the suspected allergens are the major point in the management of contact oral mucosal allergies. However, the clinical relevance assessment of haptens found positive at patch test are the main factors that make sometimes difficult the diagnosis and the management of OCA, since the avoidance of the responsible substances is arduous and not even possible and requires an interdisciplinary approach to the patient.

## Abbreviations

OCA: oral contact allergies
OLRs: oral lichenoid reactions
OLP: oral lichen planus
BMS: burning mouths syndrome
